# Understanding the Landscape of Bronchoscopy in Lung Cancer: Insights From Lesion Location, Gender, and Diagnostic Efficacy

**DOI:** 10.7759/cureus.53918

**Published:** 2024-02-09

**Authors:** Laxmi Devi, Yogendra Verma, Anand Kumar, Farhan Khan, Sanjay Verma, Ankit Kumar

**Affiliations:** 1 Respiratory Medicine, Ganesh Shankar Vidyarthi Memorial Medical College, Kanpur, IND; 2 Pathology, Ganesh Shankar Vidyarthi Memorial Medical College, Kanpur, IND; 3 Respiratory Medicine, King George's Medical University, Lucknow, IND

**Keywords:** tuberculosis, endobronchial biopsy, transbronchial needle aspiration, tbna, lung cancer, bronchoscopy

## Abstract

Introduction: Lung cancer constitutes a critical global health concern. According to the International Agency for Research on Cancer's (IARC) GLOBOCAN 2020 estimates, lung cancer is the leading cause of death in cancer patients. In areas where tuberculosis is prevalent, misdiagnosis and mistreatment frequently result from overlap, creating significant difficulties that delay diagnosis and treatment. Amid this complication, bronchoscopic techniques emerge as critical diagnostic tools, though their efficacy varies between studies.

Method: Our retrospective study, conducted from July 2021 to December 2022 at the Department of Respiratory Medicine, Ganesh Shankar Vidyarthi Memorial Medical College, Kanpur, examined 156 participants with malignancies. Our focus encompassed diverse lesions within the bronchial landscape, revealing intriguing findings.

Results: Bronchoscopic examinations unravelled prevalent abnormalities: 52 (33.3%) manifested as intraluminal growth, 48 (31.6%) as mucosal irregularities, and a less frequent (16, 10.3%) as an intraluminal bulge. Transbronchial needle aspiration stood out with a 10/11 (91%) positivity rate, biopsy came in second at 38/46 (83%), and bronchoalveolar lavage showed a 44/152 (29%) positivity rate. It was interesting to see how the lesions were spread out among the different types of histology. For example, squamous cell carcinoma showed 17/37 (46%) intraluminal growth, while adenocarcinoma showed 22/60 (36.7%) intraluminal growth and 4/60 (6.7%) intraluminal bulge. Moreover, a significant absence of abnormalities was observed in various lesions, underlining the intricacies of characterising bronchial lesions.

Conclusion: Our study shows that direct tissue sampling is better and that new bronchoscopic technologies are important for diagnosing lesions that were hard to get to in the past. However, limitations in patient selection biases and the single-centre focus caution against generalised interpretations. Our research illuminates the pivotal role of bronchoscopic methods in diagnosing lung lesions, emphasising the necessity for continued advancements to enhance diagnostic accuracy and treatment efficacy in lung cancer subtypes.

## Introduction

Lung cancer is a significant cause of death and a paramount public health concern worldwide. According to the International Agency for Research on Cancer's (IARC) GLOBOCAN 2020 estimates of cancer incidence and mortality, lung cancer continues to be the primary cause of cancer-related death, accounting for an estimated 1.8 million deaths (18%) in 2020 [1]. The leading cause of lung cancer is tobacco use, which includes using pipes, cigars, and cigarettes, but it can also occur in non-smokers [2].

In many instances, initial misdiagnosis and mistreatment of lung cancer as tuberculosis pose a significant challenge, particularly in regions where tuberculosis remains the predominant disease. This misidentification often leads to delays in diagnosing and treating lung cancer, adding complexity to its management [3,4].

The effectiveness of various bronchoscopic procedures in detecting malignancies showcases considerable variability across published studies [5]. Enhancing the detection rates of flexible bronchoscopy is crucial to addressing this diagnostic uncertainty [6].

While histopathology remains the gold standard for diagnosing lung cancer, its universal application in patients suspected of having cancer is impractical. Accessibility to bronchial biopsies proves challenging, especially in cases where lesions are located peripherally, demanding a higher level of expertise for successful retrieval [7,8].

Despite these challenges, skilled bronchoscopists utilising conventional techniques can still achieve reasonably accurate diagnoses in lung malignancy cases [9]. This introduction delves into the complexities surrounding lung cancer diagnosis, highlighting the importance of maximising detection rates in bronchoscopic procedures to combat the hurdles faced in diagnosing this lethal disease.

## Materials and methods

Study design and setting

This retrospective study was conducted at the Department of Respiratory Medicine, Ganesh Shankar Vidyarthi Memorial Medical College, Kanpur, Uttar Pradesh, India, from July 2021 to December 2022. Ethical clearance was obtained from the Institutional Review Board (Approval No.: EC/BMHR/2023/58; Reference No.: EC/263/Oct/2023) before data collection.

Participants

Patients meeting specific criteria were enrolled after obtaining written informed consent. The inclusion criteria included individuals exhibiting clinical and radiographic indications suggestive of malignancy. In contrast, exclusion criteria excluded patients with contraindications for invasive procedures, those with prior diagnoses of non-malignant respiratory conditions, and individuals unwilling to participate or provide informed consent. After screening, 156 patients were enrolled in the study.

Data collection

The retrospective data collection involved reviewing electronic health records and pathology reports of patients meeting the inclusion criteria during the designated study period.

Method

We used bronchoscopy to look into lesions in the middle of the lungs that could be reached. These were mostly checked out with bronchoalveolar lavage, biopsy, transbronchial needle aspiration (TBNA), and brushings. Also, cases with peripheral lesions where image-guided transthoracic biopsies were not possible because of a small window or because the radiologist thought they were too risky were included for bronchoscopic evaluation.

Sample collection and processing

Samples obtained through bronchoscopic procedures underwent processing following standard cytology and histology procedures. Standard protocols were promptly implemented for management when procedures were terminated due to excessive bleeding.

Documentation and analysis

Comprehensive records detailing post-procedural complications were maintained. These were diligently documented for further analysis.

## Results

Our study looked at 156 people, including 102 men (65.4%) and 54 women (34.6%), and the average age was 53 years. We used advanced bronchoscopy techniques like bronchoalveolar lavage, biopsy, TBNA, and brushings to get a better look at the bronchial lesions.

**Figure 1:**
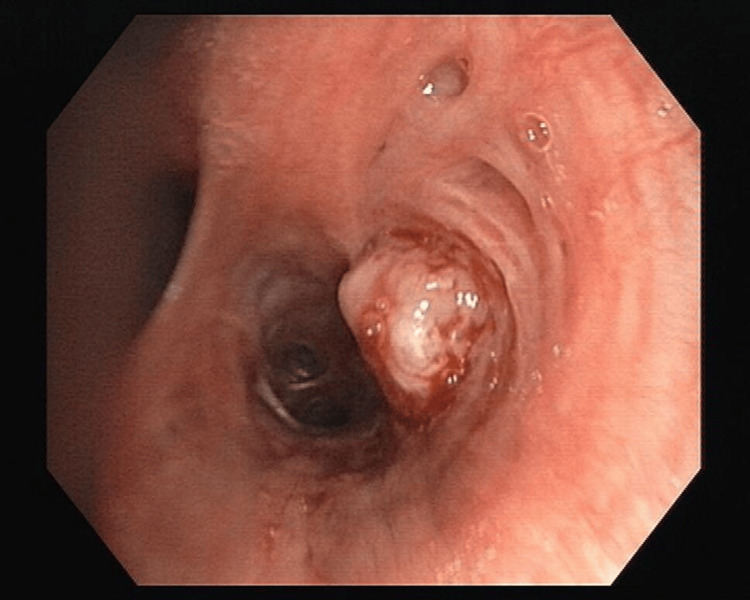
Intraluminal growth in the right lower lobe bronchus

**Figure 2:**
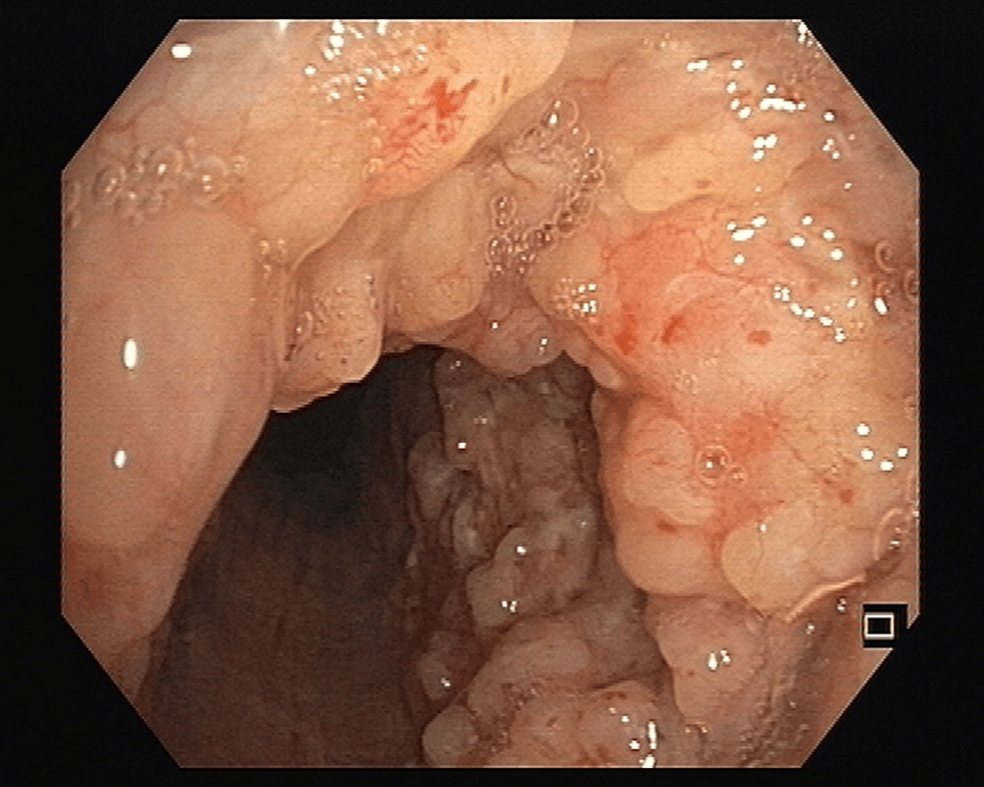
Mucosal papillomatous growth all over the main trachea

**Figure 3:**
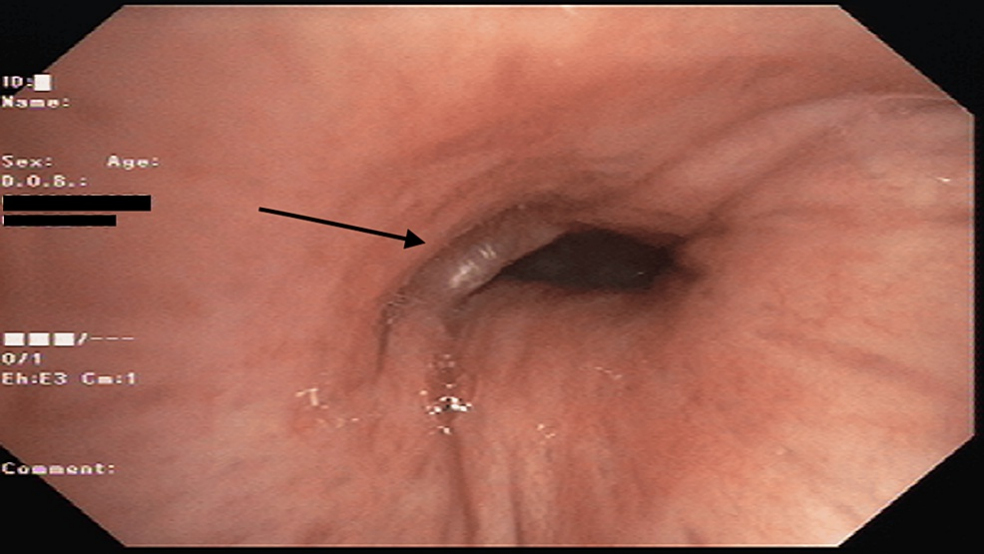
External compression at the level of left secondary carina, partially occluding left main bronchus

Among the lesions observed during bronchoscopy, the most prevalent was intraluminal growth (Figure 1), accounting for 33.3% of cases, followed by mucosal irregularities (Figure 2) at 31.6%. In contrast, intraluminal bulge (Figure 3) was the least frequent finding, representing 10.3% of the cases (Table 1).

**Table 1:** The type of lesions and their numbers Intraluminal growth was the most common finding, followed by mucosal irregularities, whereas intraluminal bulge (extraluminal growth) was the least common finding visualised on bronchoscopy.

Type of lesion	Number	Percentage (%)
No abnormality	40	25.6%
Intraluminal growth	52	33.3%
Intraluminal bulge (extraluminal growth)	16	10.3%
Mucosal irregularities	48	31.6%
Total	156	100%

**Figure 4:**
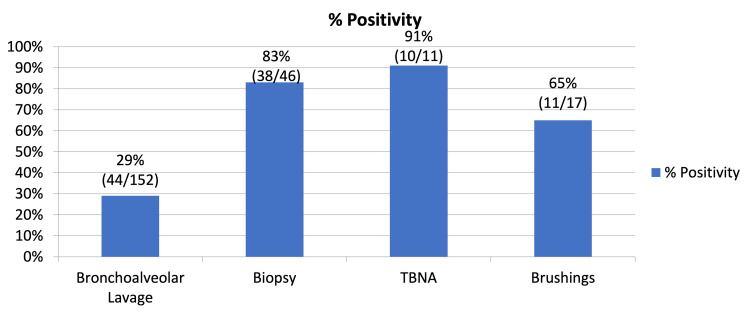
Bar graph showing diagnostic yield of different bronchoscopic procedures

TBNA demonstrated the highest positivity rate among the procedures, with 91%, followed by biopsy at 83%. Conversely, bronchoalveolar lavage exhibited a lower positivity rate at 29% (Figure 4).

Squamous cell carcinoma (SCC) accounted for 37 cases, with intraluminal growth observed in 46% and intraluminal bulge (extraluminal growth) in 13.5% of cases. Adenocarcinoma was found in 60 patients, with 36.7% showing intraluminal growth and 6.7% displaying intraluminal bulge, and small cell carcinoma was found in 20 cases, featuring a 35% incidence of intraluminal development and 20% with intraluminal bulge (Table 2).

**Table 2:** Correlation between bronchoscopic morphological appearance and histopathology

Type of lesion	Squamous cell carcinoma (37)	Adenocarcinoma (60)	Small cell carcinoma (20)	Chronic granulomatous lesion (23)	Acute on chronic inflammatory lesion(6)	Chronic inflammatory lesion (10)
No abnormality	27%	26.7%	0%	39.1%	50%	20%
Intraluminal growth	46%	36.7%	35%	17.4%	0%	20%
Intraluminal bulge (extraluminal growth)	13.5%	6.7%	20%	8.7%	0%	10%
Mucosal irregularity	13.5%	30%	45%	34.8%	50%	50%
Total	37 (100%)	60 (100%)	20 (100%)	23 (100%)	6 (100%)	10 (100%)

Chronic granulomatous lesions presented in 23 cases, with 17.4% exhibiting intraluminal growth and 8.7% showing intraluminal bulge. Chronic inflammatory lesion was found in 10 patients, with half displaying mucosal irregularities and acute on chronic inflammatory lesions were observed in six cases, predominantly with mucosal abnormalities.

Notably, no abnormalities were reported in 27% of SCC, 26.7% of adenocarcinoma, 39.1% of small cell carcinoma, 50% of chronic granulomatous lesions, 20% of acute on chronic inflammatory lesions, and 20% of chronic inflammatory lesion cases (Table 2).

## Discussion

Bronchoscopic techniques stand at the forefront of diagnosing lung lesions; their efficacy hinges on various factors like operator expertise, lesion characteristics, and visual cues during the examination [10]. Our study embarked on a journey to explore these nuances, revealing pivotal insights into diagnosing lung cancer within the larger airways.

Using bronchoscopic techniques, we examined the complex terrain of bronchial lesions in 156 participants, 102 male (65.4%) and 54 female (34.6%), with a mean age of 53 years.

The diagnostic accuracy of our methods was clear, showing that TBNA and biopsy worked, with success rates of 91% and 83%, respectively. In contrast, bronchoalveolar lavage exhibited a lower positivity rate at 29%. The herald of diagnostic accuracy emerged as TBNA, which had the highest percentage of positive samples, with biopsy coming in second. At the same time, bronchoalveolar lavage, although invaluable, showcased a lower positivity rate [11].

Our study revealed intriguing complexities in bronchial lesions. While SCCs predominantly grew within the airways, adenocarcinomas displayed diverse patterns. Notably, a significant number across various types lacked reported abnormalities, highlighting limitations in current diagnostic methods. These "invisible" lesions suggest underlying intricacies demanding more sophisticated approaches. To optimise patient care, we need continuous advancements in diagnostic techniques, multidisciplinary evaluation, and further research to understand lesion presentation and develop better strategies. This will ultimately enhance diagnostic accuracy, improve patient care, and advance our understanding and management of bronchial pathology [12,13].

Comparing our study to previous research by Rabahi et al., intriguing disparities emerged in the patterns of bronchial lesions and their endoscopic presentations. While Rabahi et al. emphasised endobronchial masses and mucosal infiltration as primary findings, our investigation unveiled intraluminal growth as the predominant lesion, closely followed by mucosal irregularities, with intraluminal bulges being less frequent. It was clear that these patterns were different for different types of histology. For example, SCC mainly showed intraluminal growth, while adenocarcinoma had fewer cases of intraluminal bulge. Such variances underscore the importance of recognising specific patterns for accurate diagnosis and tailored treatment strategies in bronchial lesions [14].

In contrast, the comparative analysis with Biciuşcă et al.'s study highlighted intriguing differences in our observations and the abnormalities encountered. Using flexible fiberoptic bronchoscopy (FFB), our study found that intraluminal growth within the airway was the most common abnormality, seen in about a third of cases. Surface irregularities came right after this. Astonishingly, extraluminal growth, bulging from the airway walls outward, was the least frequent finding in only about 10% of cases. Exploring various lung cancer types, such as SCC, adenocarcinoma, and small cell carcinoma, revealed distinctive growth patterns within or outside the airways. SCC primarily showed growth within the air passage, whereas adenocarcinoma exhibited intraluminal and extraluminal growth patterns, shedding light on the diverse visual appearances during bronchoscopy [15].

The comparison with studies by Leong et al. (2013) and Choudhuri et al. (2020) highlights distinct lesion characteristics and variations in reporting abnormalities. In our study, SCC was found to be common and adenocarcinoma showed unique intraluminal growth. However, a lot of cases across all lesions did not have any reported abnormalities. Despite differences in methodologies, these findings guide clinical decision-making and diagnostic strategies, emphasizing the need for further investigation to enhance accuracy in bronchoscopic evaluations [16,17].

Our research confirms the superiority of direct tissue sampling methods like biopsy and TBNA over indirect techniques such as bronchial lavage, aligning with previous studies. Despite bronchial lavage's lower diagnostic yields, its safety and simplicity warrant its continued use. Additionally, advancements in scopes have improved the detection of centrally located adenocarcinomas, reflecting the evolving landscape of bronchoscopic diagnosis. Integrating both direct and indirect methods remains crucial for comprehensive evaluations, optimizing patient care amidst technological advancements [16,17].

The most common complications observed in our study were haemoptysis and hypoxia, highlighting the clinical significance and potential risks associated with bronchoscopic procedures. Furthermore, the identification of haemoptysis and hypoxia as common complications in our study emphasises the need for comprehensive pre-procedural assessment and risk stratification to identify patients at higher risk for adverse events. Close collaboration between bronchoscopists, anaesthesiologists, and respiratory therapists is essential to optimise patient care and minimise the occurrence and severity of complications during bronchoscopic procedures [3,6].

Despite these critical insights, our study grapples with limitations. Potential biases in patient selection skew findings toward specific lesion types or diagnostic outcomes. Additionally, the focus on a single medical centre might limit the broader applicability of our results to diverse populations or healthcare settings.

## Conclusions

Our study echoes the importance of direct tissue acquisition methods in augmenting diagnostic accuracy for lung lesions. While bronchial lavage might not exhibit high diagnostic rates, its inclusion remains justifiable due to safety, ease of execution, and potential to uncover alternative diagnoses. The strides made in bronchoscopic technology mark a promising shift in diagnosing lung cancer subtypes, particularly adenocarcinomas, which are traditionally challenging due to their peripheral nature. These advancements signal a bright future in diagnostic capabilities, emphasising the imperative role of evolving bronchoscopic techniques in clinical practice.
